# Evaluation of a microwave based reactor for the treatment of blackwater sludge

**DOI:** 10.1016/j.scitotenv.2016.01.013

**Published:** 2016-04-01

**Authors:** Peter M. Mawioo, Audax Rweyemamu, Hector A. Garcia, Christine M. Hooijmans, Damir Brdjanovic

**Affiliations:** a Department of Environmental Engineering and Water Technology, UNESCO-IHE Institute for Water Education, Westvest 7, 2611 AX Delft, The Netherlands; b Department of Biotechnology, Delft University of Technology, Julianalaan 67, 2628 BC Delft, The Netherlands

**Keywords:** Emergency sanitation, Blackwater, Fecal sludge, Microwave irradiation, Volume reduction, Pathogen reduction

## Abstract

A laboratory-scale microwave (MW) unit was applied to treat fresh blackwater sludge that represented fecal sludge (FS) produced at heavily used toilet facilities. The sludge was exposed to MW irradiation at different power levels and for various durations. Variables such as sludge volume and pathogen reduction were observed. The results demonstrated that the MW is a rapid and efficient technology that can reduce the sludge volume by over 70% in these experimental conditions. The concentration of bacterial pathogenic indicator *E. coli* also decreased to below the analytical detection levels. Furthermore, the results indicated that the MW operational conditions including radiation power and contact time can be varied to achieve the desired sludge volume and pathogen reduction. MW technology can be further explored for the potential scaling-up as an option for rapid treatment of FS from intensively used sanitation facilities such as in emergency situations.

## Highlights

• There is lack of fast and efficient fecal sludge treatment options in emergencies.
• Microwave treatment is rapid and efficient in sludge volume and pathogen reduction.
• Power and contact time can be varied to reach diverse levels of sludge treatment.

## Graphical abstract

**Figure:**
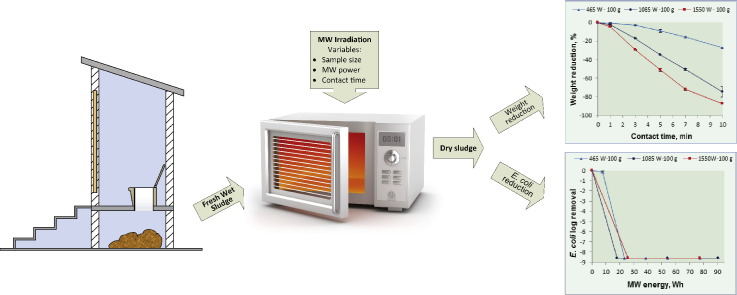

## 1 Introduction

Heavy usage of onsite sanitation facilities (i.e. 50–400 users per sanitation facility as observed in emergency settings) results in the rapid accumulation of fresh fecal sludge (FS) which should be frequently and safely disposed. Rapid accumulation rates result in the generation of large volumes of FS which can create a significant challenge for FS management especially, during its transportation and disposal. The situation can be worsened by the generation of such huge amounts of FS in a locality with limited disposal possibilities. Particularly, if the availability of land is inadequate for local disposal, the FS may need to be hauled long distances to the final disposal site. In such situations, it might be economical to reduce the sludge volume in order to minimize the transport and ultimately the operation and maintenance costs of the sanitation system. Furthermore, FS contains various compounds of interest including high concentrations of organic and inorganic matter, and large amounts of pathogens. Pathogenic organisms found in FS include bacteria, viruses, protozoa, and helminths (WHO, 2001, Fidjeland et al., 2013). These organisms present a major concern to public health especially in the disposal and/or reuse of the sludge. The pathogenic organisms should thus be reduced to safe levels (e.g. *E. coli* ≤ 1000 CFU/g TS (WHO, 2006)) in order to minimize public health risk posed by the possible outbreaks of excreta related epidemics. The presence of organic matter in the FS is yet another important aspect in the FS management as it can lead to offensive odors and attract vector organisms (such as houseflies and mosquitoes) that can spread diseases. Therefore, organic stabilization of the FS is desirable to ensure safe waste disposal practice. The aspects highlighted above form a major challenge in FS management but can be addressed by the use of appropriate FS treatment technologies.

A number of FS conventional treatment options are available, including the conventional drying (e.g. in sludge drying beds), composting, co-composting with organic solid waste, anaerobic co-digestion with organic solid waste (producing biogas), and co-treatment in wastewater treatment plants (WWTP) (Ingallinella et al., 2002, Ronteltap et al., 2014). These technologies have been tested and applied in regular sanitation contexts. They have associated benefits, but also limitations that make their application less suitable in some specific contexts such as the emergency situations. For instance, the composting technology produces a hygienically safe product rich in humus; however, it requires much space, long treatment duration, and may pose environmental pollution and public health risks in low-lying areas in the case of flooding (Katukiza et al., 2012). Anaerobic co-digestion with organic solid waste offers the benefit of both increasing biogas production, as well as using the final (end) product as a fertilizer. However, this option has the limitation of involving a relatively slow digestion process. In addition, a post treatment stage is required for the further removal of pathogens (Katukiza et al., 2012). Co-treatment in WWTPs is a possible option for FS treatment, but the probable heavy hydraulic, organic, and solids loads may limit the application of this alternative (Lopez-Vazquez et al., 2014), especially, if the co-treatment of sludge was not considered in the original design.

Generally, the major limitations of conventional FS treatment technologies highlighted above include their relatively slow treatment processes and large space requirements, which make them less feasible in scenarios with high FS generation rates and limited land space. Such scenarios are commonly faced when dealing with heavily used onsite facilities such as during an emergency situation. Inappropriate FS treatment in emergencies has, in some cases, resulted in the adoption of poor disposal methods, especially in less developed countries where common practice is to use pit latrines and septic tanks. These facilities fill up rapidly when intensively used, in which case the adoption of poor disposal methods is likely to occur. A recent example is the open dumping of raw FS reported in Haiti after the earthquake in 2010 (Oxfam, 2011). Such poor FS management practices pose great dangers to affected people whose public health is already jeopardized by the poor living conditions in the disaster environment. For instance, the rapid spread of a cholera epidemic after the Haiti earthquake in 2010 which claimed approximately 500,000 lives was associated with inadequate sanitation provision (Tappero and Tauxe, 2011). Similarly, sanitation related outbreaks of diarrheal diseases were reported after the earthquake in Pakistan in 2005, the tsunami in Indonesia in 2004, the floods in Bangladesh in 2004, and the floods in Mozambique in 2000 (Watson et al., 2007).

These challenges for FS treatment in areas with high generation rate and limited land space demonstrate the need to explore more options which are fast and efficient. MW technology may present an appropriate alternative for future applications in FS treatment in such areas. The MW irradiation has characteristics such as instant and accurate control of the power input as well as providing fast and uniform heating throughout the target material (Haque, 1999). With its unique nature in rapid heating, the MW technology application appears very promising for FS treatment in the situations requiring rapid treatment options. Furthermore, MW based applications can potentially provide compact and easily portable as well as fast and effective FS treatment package units with reduced footprints. The MW technology uses microwave energy which is a non-ionizing electromagnetic radiation with wavelengths between 1 mm and 1 m and frequencies between 300 MHz and 300 GHz (Haque, 1999, Tang et al., 2010, Remya and Lin, 2011). The technology has been widely used in communication, industrial, scientific, medical and instrumentation applications (Haque, 1999). Most of the above applications utilize the technology for heating where the microwaves cause molecular motion in the target material by inducing the migration of ionic species and/or the rotation of dipolar species (Haque, 1999, Thostenson and Chou, 1999). The heating by microwaves depends on the dissipation factor, which is the ratio of the dielectric loss factor to the dielectric constant of the target material. The dielectric loss factor depicts the amount of MW energy lost in the material by dissipation in form of heat while the dielectric constant depicts the ability of material to delay or retard microwave energy as it passes through. Therefore, materials that are easily heated by MW energy have a high dielectric loss factor (Haque, 1999, Thostenson and Chou, 1999).

The MW technology was applied in the treatment of some common wastes such as sewage sludge which contains dipolar molecules (e.g. water and organic complexes) with high loss dielectric properties that enable selective and concentrated heating by microwaves (Yu et al., 2010). A number of studies that were conducted using the various types of sewage sludge demonstrated the success of MW treatment in many aspects including pathogen reduction (Tyagi and Lo, 2013). For instance, complete pathogen destruction was reported when primary, anaerobic digester and waste activated sludges were heated by MW to temperatures above 65 °C (Hong et al., 2004, Hong et al., 2006, Tyagi and Lo, 2013). In addition, over 80% volume reduction was reported when anaerobic sewage sludge was exposed to MW irradiation (Menéndez et al., 2002). The pathogen destruction by MW was associated with both the non-thermal (electromagnetic radiation) and thermal (temperature) effects of the electromagnetic energy. Electromagnetic radiation causes the molecules of the irradiated material to orient themselves in the direction of the electric field. This may result in hydrogen bonds breaking leading to the denaturation and death of microbial cells (Banik et al., 2003, Tyagi and Lo, 2013). On the other hand, the destruction by thermal effect is caused by the rotation of dipole molecules under an oscillating electromagnetic field which results in rapid heating of water to boiling point. The cells of microorganisms are then ruptured and the bound water is released (Hong et al., 2004, Wojciechowska, 2005, Tang et al., 2010, Tyagi and Lo, 2013).

Such successful applications in sewage sludge treatment demonstrate the potential of the MW technology in treating FS. Although different in aspects such as concentration of organics, pathogen, TS, and others, the fresh FS has relatively similar dielectric properties to that of sewage sludge. For instance, FS contains dipolar (e.g. water and organic complexes) molecules which are important in the MW heating. The dipolar characteristic of the fresh FS thus provides an opportunity for its treatment by the MW technology.

Despite the successful evaluations of the MW treatment using various kinds of sewage sludge, no studies have yet been reported with respect to FS context. It is thus desired to evaluate MW application on FS, since FS is more concentrated, and comparatively has more pathogens, and organics, among others. Therefore, the objective of this study is to investigate the potential of a microwave (MW) based technology for treating fresh blackwater FS extracted from a highly concentrated domestic blackwater stream. The study focused on three aspects of treatment namely the volume reduction (drying), sanitization (bacterial pathogen reduction), and organic stabilization (organic matter reduction) in the sludge. The weight was used to estimate the volume reduction while the removal efficiency of *E. coli* was used as an indicator for the reduction of bacterial pathogens. The volatile and total solids ratio (VS/TS) was used as an indicator for the organic stabilization of the sludge. This is arguably the first study to evaluate the use of MW technology for fresh FS treatment. If successful, this technology may provide a solution to the complex task of FS treatment; particularly, when dealing with heavily used onsite sanitation facilities such as in emergency settings.

## 2 Materials and methods

### 2.1 Research design

This study was performed using blackwater FS which was extracted by centrifugation from autoclaved and non-autoclaved highly concentrated fresh blackwater stream obtained from the DESAR (Decentralized Sanitation and Reuse) demonstration site in Sneek (Friesland, NL). The 20 g sample was autoclaved to remove all the existing organisms in the sludge, and then a known concentration of harmless *E.coli* was introduced and its response to MW treatment was closely observed. Then the study was advanced with the non-autoclaved 100 g sample in which the *E.coli* naturally occurring in the sludge was monitored. Being a type of FS (Strande, 2014), blackwater could be directly used in this study. However, the aim was to evaluate the MW treatment with a more concentrated FS stream, similar to what is generated in emergency situations where non-flush toilet facilities are commonly applied. And since this kind of FS could not be obtained in The Netherlands, as the country is mostly sewered, the centrifugation of blackwater to obtain a more concentrated FS was considered. Two sludge fractions (i.e. 20 g with the autoclaved sludge and 100 g with the non-autoclaved sludge) were treated in a domestic MW oven for various durations and input MW power levels. Changes in temperature, weight reduction (volume indicator), *E. coli* (pathogen indicator) and VS/TS ratio (organic matter indicator) were measured in the samples treated by MW. The experiments using the 20 g sludge were conducted in duplicate while those using 100 g sludge were conducted in triplicate. The effectiveness of the MW treatment was evaluated based on changes in the measured parameters before and after exposure to MW.

### 2.2 Microwave apparatus

A domestic MW oven, Samsung, MX245 (Samsung Electronics Benelux B.V., The Netherlands) was used in this study. The unit operates at a frequency of 2450 MHz with a power output ranging from 0 to 1550 W with 10% incremental steps.

### 2.3 Sludge samples

The blackwater samples were drawn from a buffer tank receiving blackwater collected from vacuum toilets flushed with approximately 0.5 L of water per use. The samples were then transported to the research laboratory and stored at 4 °C prior to the experiments within 48 h. The proximate characteristics of the fresh blackwater are presented in Table 1. The blackwater was concentrated by centrifugation to attain a blackwater FS with proximate characteristics as presented in Table 1, which are common for FS from non-flush toilet facilities (Fidjeland et al., 2013).

### 2.4 Experimental procedures

#### 2.4.1 Sample preparation

The characteristics of the fresh blackwater FS sample that was applied in this study are presented in Table 1 above. The blackwater used to prepare the 20 g samples was autoclaved at 121 °C for 1 h in a standard autoclave (Tuttnauer, model 3870 ELV, Tuttnauer Europe B.V., Breda, The Netherlands) to destroy all existing organisms. The sample was then concentrated using a bench-top centrifuge (ROTINA 420, Hettich, Germany) operated at a relative centrifugal force (RCF) of 1851 for 30 min. Following this, the supernatant was discarded and the resulting sludge cake (i.e. sludge cake, TS = 12%) was spiked with a harmless *E. coli* (type ATCC25922) to the final concentration of 10^8^ CFU/g TS. The test samples for the MW treatment were prepared in duplicates by placing 20 g samples (height approximately one centimeter, and surface area approximately 33.2 cm^2^) in a 250 mL glass beaker. Similarly, the 100 g samples were prepared in the same procedure explained above but with non-autoclaved fresh blackwater. The resulting sludge cake (for the non-autoclaved 100 g samples) was not spiked as it contained the *E. coli* naturally occurring in human excreta (10^8^ CFU/g TS, TS = 12%). The test samples for the MW treatment were prepared in triplicates by placing 100 g samples (height approximately one centimeter, and surface area approximately 78.5 cm^2^) in a 1000 mL glass beaker. In both cases, *E. coli* was used as an indicator to evaluate the MW capability to destruct the pathogenic bacteria in the sludge.

#### 2.4.2 Microwave treatment

Both the 20 g (autoclaved) and 100 g (non-autoclaved) sludge samples were treated using the MW apparatus described in the Section 2.2 above. The sample contained in the glass beaker was placed in the MW cavity and exposed to the MW irradiation at 465, 1085, and 1550 W for varied time durations. The 20 g samples were exposed for 5, 10, 20, 30, 60, 120, 180, and 240 s (i.e. 0.08, 0.17, 0.33, 0.5, 1, 2, 3, and 4 min, respectively), while the 100 g samples were exposed for 1, 3, 5, 7, and 10 min. After the MW treatment, the sample was removed from the MW cavity and its temperature was immediately measured before covering with sanitized aluminum foil. The microwaved samples were cooled to room temperature and analyzed for their characteristics as described in the following section.

### 2.5 Analytical procedures

Sludge samples with and without MW treatment were measured for physical, chemical and microbial parameters including the total COD (TCOD), temperature, weight/volume reduction, TS, VS, and *E. coli*.

#### 2.5.1 COD measurement

Samples for COD measurement in the blackwater (prior to centrifuging) and the blackwater FS (prior to MW treatment) were prepared by diluting a known amount in demineralized water. The COD measurement was then done according to the closed reflux method (SM 5220 C) as outlined in the Standard Methods for the Examination of Water and Wastewater (APHA, 1995). The values were expressed in mg COD per g TS (mg COD/g TS) as shown in Table 1 (Section 2.3).

#### 2.5.2 Temperature measurement

The initial sample temperature was measured just before MW irradiation using an infrared thermometer (Fluke 62 MAX, Fluke Corporation, U.S.A). Following each treatment, a sample was taken from the MW cavity and the final temperature was immediately measured. While measuring the final temperature, samples were mixed by shaking to avoid taking only temperature at the surface of the heated sludge. However, for those samples that were too dry to mix by shaking (TS ≥ 17%), the temperature was measured on the surface.

#### 2.5.3 Weight measurement

Samples weight measurements were done using a bench-top weighing balance (Sartorius H160, Sartorius AG, Germany). The initial weight was measured as the samples were transferred into the heating beakers, while the final weight was measured once the samples were cooled to room temperature after the MW treatment. The volume reduction was then determined from the difference between the initial and the final sample weight. Based on the maximum temperature attained during MW treatment (i.e. ≤ 127 °C), the weight reduction could only be attributed to the water evaporating from the heated sludge. Thus, considering the density of water, the weight reduction was deemed to be equivalent to the sludge volume reduction.

#### 2.5.4 Microbial measurement

The detection of *E. coli* was done using the surface plate technique with chromocult coliform agar (Chromocult; Merck, Darmstadt, Germany) (Byamukama et al., 2000). Portions of one gram from each MW treated sample and the control (untreated sludge) were transferred to sanitized plastic containers (20 mL), then mixed with 9 mL of buffered peptone water and thoroughly homogenized. A potter tube (Potter-Elvehjem PTFE pestle and glass tube, Sigma-Aldrich Co. LLC, USA) was used to grind samples that were too dry to directly dissolve in the buffered peptone water. The homogenized samples were serially diluted (10^− 1^ to 10^− 5^) with the buffered peptone water. Partitions of 100 μL of the respective sample dilutions were applied to the chromocult agar plates in duplicate for each dilution step. All plates were then incubated at 37 °C for 24 h, after which the average numbers of colonies in plates were counted. The counting was visually facilitated by a colony counter (IUL magnifying glass colony counter, IUL, S.A., Barcelona, Spain). Dark blue to violet colonies were classified as *E. coli* (Byamukama et al., 2000, Sangadkit et al., 2012). The average number of colonies was used to calculate the viable-cell concentrations in the solid samples, expressed in CFU/g TS of the test sample.

#### 2.5.5 TS and VS measurement

For each treated sample the TS and VS values were measured according to the gravimetric method (SM-2540D and SM-2540E) by drying a known sample weight in an oven at 105 °C for 2 h (for TS) and subsequently in a muffle furnace at 550 °C for 2 h (for VS) as outlined in the Standard Methods for the Examination of Water and Wastewater (APHA, 1995). The TS and VS results were then used to evaluate sludge stability based on the organic matter reduction.

## 3 Results

### 3.1 Temperature evolution

**Fig. 1:**
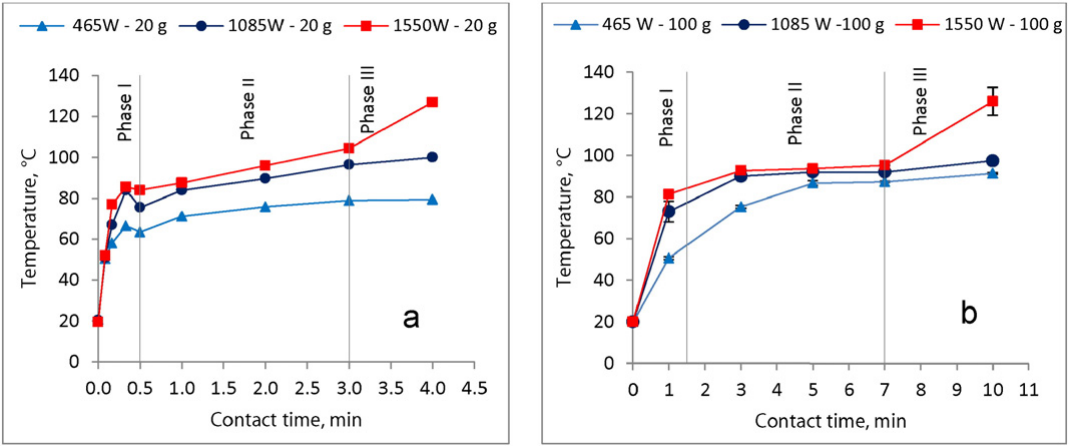
Effect of exposure to MW irradiation on temperature in the a) 20 g sludge sample and b) 100 g sludge sample.

Fig. 1a and b show the temperature profiles during MW exposure of the 20 g and 100 g sludge sample, respectively. As expected, the temperature increment rate increased as the MW power input rose, with 1550 W inducing the highest rate. As shown in Fig. 1a and b, three phases were observed in the temperature rise during the MW heating. The first phase shows a rapid increase in sludge temperature, the second phase shows a fairly constant and minimal temperature rise, and the third phase depicted in again a rapid temperature increase. However, in this case the third phase was only achieved when the sludge was heated at 1550 W at contact time above 3 and 7 min for the 20 g and 100 g samples, respectively. This implies that for the 465 W and 1085 W, contact times longer than 4 min for 20 g and 10 min for 100 g are required to attain the third temperature phase.

### 3.2 Volume reduction and energy requirements

**Fig. 2:**
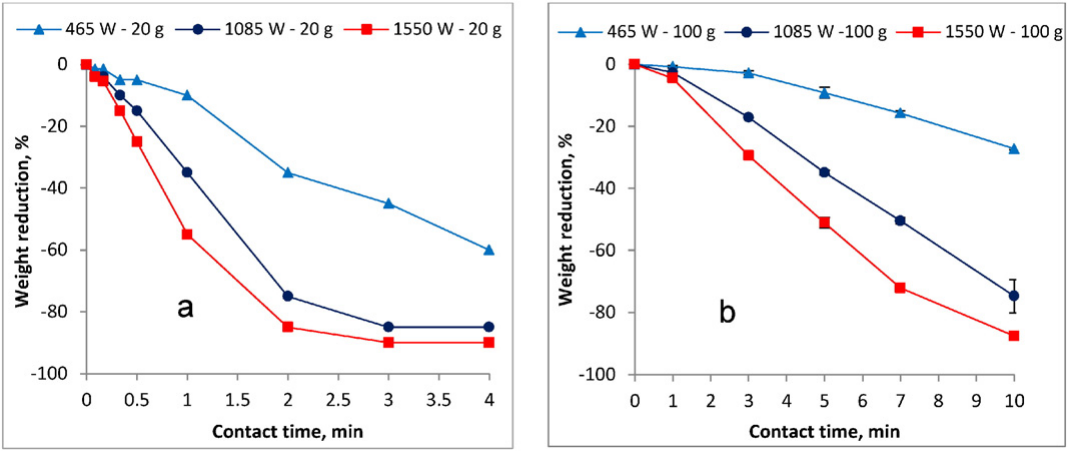
Effect of exposure to MW irradiation on sludge weight in the a) 20 g sludge sample and b) 100 g sludge sample.

Fig. 2a and b show the respective weight/volume reduction when the 20 g and 100 g sludge samples were exposed to different MW power input levels and contact times. The weight/volume reduction is mainly associated with the temperature changes and the resulting moisture loss in the irradiated sludge. The weight/volume reduction process seems to occur in three stages which closely follow the trend of the three temperature phases described in Section 3.1 above. These drying stages were identified as the preliminary, essential (major), and final drying phases (Flaga, 2005). In the 20 g sample size (as illustrated in Fig. 2a), the preliminary drying phase occurred within the first 10 s for the 1085 W and 1550 W power levels corresponding to 4 and 5.5% volume reduction, respectively. However, this preliminary drying phase lasted longer, until 30 s at 465 W with approximately 5% weight/volume reduction. Immediately after the preliminary phase the treatment entered the essential (major) drying phase depicted by high and relatively constant moisture evaporation rates. The duration for the essential drying phase varied with the power input level. For instance, the essential drying phase lasted until approximately 2.5 and 2 min for the 1085 and 1550 W, respectively, while this phase was not conclusively achieved at the 465 W in the range of the contact times evaluated in these experiments. The final drying phase was achieved only with the 1085 and 1550 W power inputs and depicted by the lowest observed sample weight/volume reduction. Similarly, the weight/volume reduction profiles for the 100 g samples were observed (as illustrated in Fig. 2b) and the trends were comparable to that reported on the 20 g sample. For instance, the preliminary drying phase lasted for approximately 1 min (60 s) in the 1085 W and 1550 W power input levels with approximate weight/volume reductions of 2.7 and 4.5%, respectively. Furthermore, at 465 W power input level, the preliminary drying phase extended to approximately 3 min (180 s) with 2.8% weight/volume reduction. Moreover, in a trend similar to that observed in the 20 g sample, the duration of the entire essential (major) drying phase in the 100 g sample varied with the power input. This phase lasted until 7 min (420 s) when the sample was irradiated at 1550 W, but it was not conclusively achieved at 465 and 1085 W in the range of contact times evaluated in these experiments. High weight/volume reductions (over 80%) were achieved for both sample sizes in which a big fraction of the weight/volume reduction is associated with the major (essential) drying phase.

**Fig. 3:**
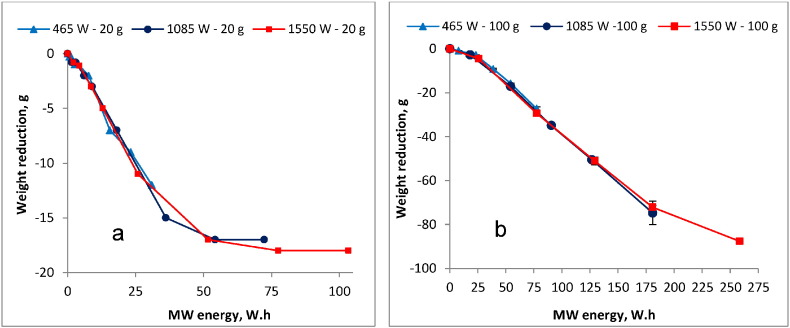
Sludge weight reduction and MW energy demand in the a) 20 g sample and b) 100 g sample.

Furthermore, the energy consumption profiles during the MW heating were observed for both the 20 g and 100 g samples and they are presented in Fig. 3a and b, respectively. The trends depicted in the energy consumption profiles correspond with the preliminary, essential and final drying phases that were previously discussed in this section.

A linear regression was performed on each of the three drying phases to reveal the corresponding specific energy demand rates in both the 20 g and 100 g sample. It was observed that for the 20 g sample, one watt-hour (Wh) was required to remove approximately 0.25 g (i.e. approximately 4 kWh per kg) during the preliminary drying. In the essential drying phase, where the lowest energy demand was observed, 1 Wh was required to achieve approximately 0.4 g reduction (i.e. approximately 2.5 kWh per kg). The final drying phase marked by relatively high energy input with low corresponding weight/volume reduction was achieved only at the 1550 W power input level, where 1 Wh was required to remove approximately 0.21 g (i.e. approximately 4.8 kWh per kg). It was also observed that the specific energy demand rates in the 100 g sample showed similar trends to that observed in the 20 g sample. In this case, 1 Wh was required to remove approximately 0.12 g (i.e. approximately 8.3 kWh per kg), which was almost twice the energy demand compared to that in the 20 g sample (i.e. 4 kWh per kg), during the preliminary drying phase. However, in the 100 g sample, the energy demand during the essential drying phase in which 1 Wh was required to remove approximately 0.44 g (i.e. approximately 2.3 kWh per kg), was relatively similar to that required in the 20 g sample (i.e. 2.5 kWh per kg) under the same drying phase.

### 3.3 Bacterial reduction

**Fig. 4:**
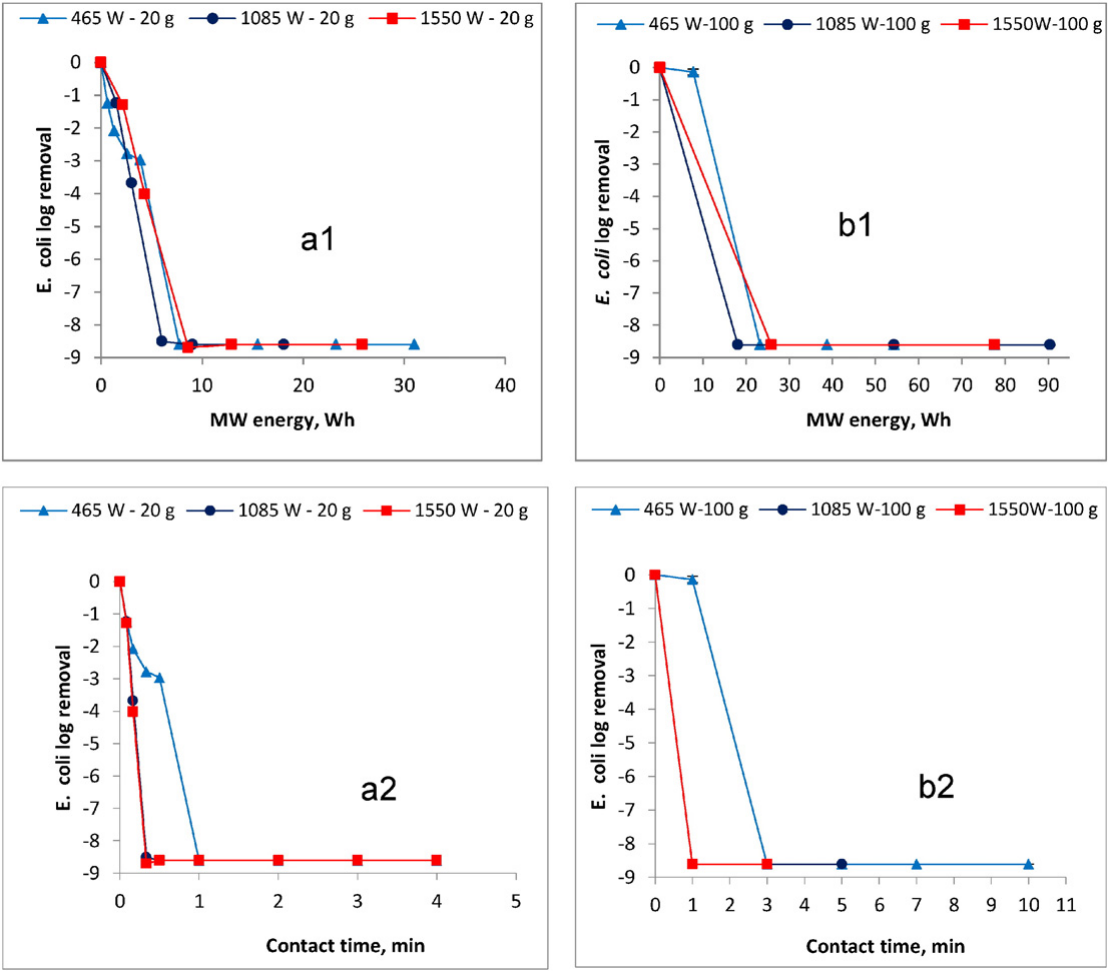
Effect of MW energy on *E. coli* reduction in a1) 20 g sludge sample and b1) 100 g sludge sample, and *E. coli* reduction as a function of time in a2) 20 g sludge sample and b2) 100 g sludge sample. The zero *E. coli* log removal corresponds to an initial concentration of 4.0 × 10^8^ CFU/g TS).

Reduction results of the *E. coli* in the 20 g and 100 g sample obtained at various MW power input levels and contact times are shown in Fig. 4a1 and a2, and b1 and b2, respectively.

**Fig. 5:**
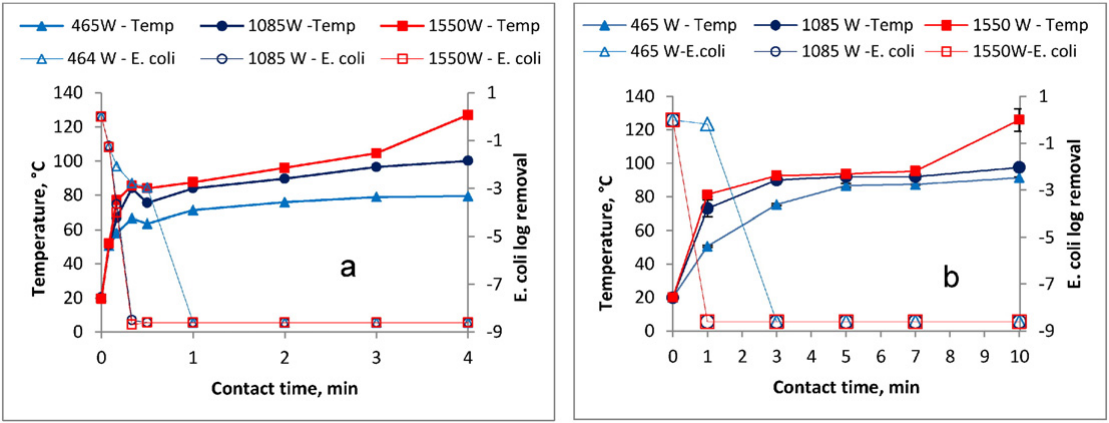
Influence of temperature on *E. coli* reduction in a) 20 g sludge sample and b) 100 g sludge sample. The zero *E.coli* log removal corresponds to an initial concentration of 4.0 × 10^8^ CFU/g TS).

Furthermore, the influence of temperature on the *E. coli* reduction over contact time in the 20 g and 100 g sample is illustrated in Fig. 5a and b, respectively.

The results show that the increases in MW power input and/or contact time (see Fig. 4a1, a2 and b1, b2) and the sludge temperature (see Fig. 5a and b) led to increased *E. coli* reduction. For instance, in the 20 g sample, an *E. coli* reduction of approximately 3 log removal value (LRV) was achieved when the sludge was MW treated at 465 W over a 0.5 min (30 s) contact time (i.e. MW energy = 4 Wh, temp. = 63 °C). However, reduction below detection limit (i.e. < 1000 CFU/g TS) was achieved with 1 min contact time (i.e. MW energy = 8 Wh, temp. = 71 °C). The exposure time for reduction to below the detection limit was shorter at 0.5 min (30 s) (i.e. MW energy = 6 Wh, temp. > 70 °C) when the sludge was irradiated at higher MW power levels equal to or higher than 1085 W. Similarly, while the MW treatment at 465 W for 1 min (MW energy = 8 Wh, temp. = 51 °C) depicted the lowest *E. coli* reduction (approximately 0.2 LRV) in the 100 g sample, a reduction to below the detection limit was realized at the same power level when the contact time was increased to 3 min (MW energy = 23 Wh, temp. = 75 °C). However, *E. coli* reduction below detection limit could still be achieved at 1 min when higher MW power input levels (e.g. 1085 W, MW energy = 18 Wh, temp. = 73 °C and 1550 W, MW energy = 26 Wh, temp. = 81 °C) were used.

### 3.4 Effect of microwave irradiation on organic stabilization of sludge

**Fig. 6:**
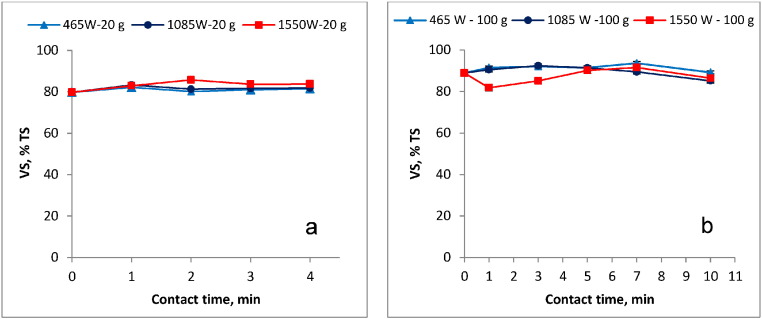
VS/TS variation in the a) 20 g and b) 100 g MW treated sludge sample.

The VS/TS ratio in the treated samples was used as an index to determine the organic stability of the blackwater FS. Fig. 6a and b show the variation in the VS/TS ratio as a function of MW power and contact time in the 20 g and the 100 g samples, respectively. As shown in Fig. 6a and b, the MW treatment was not successful in organic matter reduction as there was no significant change in the VS/TS ratio in the treated sludge. For instance, the respective initial VS/TS ratios in the 20 g and 100 g untreated samples was approximately 80% and 89%, while the final VS/TS ratio attained at all power input levels and contact times evaluated were in the same range.

## 4 Discussion

### 4.1 Temperature evolution

Results from Fig. 1a and b show that MW treatment was very effective and fast in raising the temperature in the blackwater FS. The temperature evolution in both the 20 g and 100 g sample was depicted in three phases classified as the preliminary, essential (major), and final drying phases. These temperature evolution phases conform to those reported in other drying methods such as convection and conduction (Flaga, 2005, Bennamoun et al., 2013). However, Bennamoun et al. (2013) gave a different terminology for the three phases, namely the adaptation phase, constant drying rate phase, and falling drying rate phase. The trends observed in this study also agree with those from previous studies involving MW heating of different kinds of sewage sludge (Menéndez et al., 2002, Hong et al., 2004, Yu et al., 2010, Lin et al., 2012). Furthermore, the results show that the heating rate increases with the MW power input increment which is similar to the trend reported in other studies (Eskicioglu et al., 2007, Lin et al., 2012) when sewage sludge was heated at varied MW power input levels. This can be explained by the resulting MW energy which is a function of the power input.

The rapid rise in temperature observed during the first (preliminary) drying phase can be attributed to the interaction between the microwaves (i.e. high frequency electromagnetic radiation) with the dipolar molecules of high loss dielectric properties (e.g. water, proteins, etc.) that are initially present at high concentrations in the wet blackwater FS. This interaction causes the molecular rotation resulting in the rapid heating of the sludge (Yu et al., 2010). As expected, the temperature increased more rapidly in the 20 g samples than in the 100 g samples. This can mainly be attributed to the different amounts of water content in the different sample sizes. Water has a high thermal capacity, thus by virtue of its higher water content, the bigger sample size (100 g) has a higher capacity to absorb a bigger fraction of the initial MW energy. This occurs with a relatively smaller temperature increase rate than in the smaller sample size (20 g). Similar observations were made by Tang et al. (2010) when excess sewage sludge was heated with different water contents. In the second (essential/major) drying phase, the sludge may have reached boiling point as it was characterized by a fairly constant and minimal rise in temperature. At this stage the unbound water evaporates from or near the surface of the sludge particles at constant rate. The sludge particles are covered by water on the surface that constantly evaporates as it is replaced by water from inside the particles (Flaga, 2005). As the unbound water is depleted, the heating entered the third (final) drying phase in which the sludge temperature begun to rise rapidly. According to Flaga (2005), in this phase, water on the surface of the particles evaporates faster than it is replaced by water from inside the particle. In this study, the final drying phase was only realized when sludge was heated at 1550 W at contact time above 3 and 7 min for the 20 g, and 100 g samples, respectively. That is, the final drying phase was not attained at 465 W and 1085 W demonstrating that longer contact time is required once the MW power input level is reduced. This is also true for the bigger samples that need more contact time to achieve similar results as in the smaller samples. If heating is continued in the final drying phase, it appears that the temperature rise reaches a certain maximum after which there is hardly any significant temperature increase. For instance, Menéndez et al. (2002) reported a maximum temperature of approximately 200 °C with MW heating of sewage sludge. However, the maximum attainable temperature varies from material to material as was demonstrated when a MW receptor material was mixed with sewage sludge to raise the maximum temperature to over 900 °C (Menéndez et al., 2002, Menéndez et al., 2005).

The results of this study show that temperature evolution for the MW heated blackwater FS follows similar trend as observed in sewage sludge when heated either by MW or conventional thermal technologies. Furthermore, as confirmed in several studies that compared MW to the conventional heating (e.g. water bath (Hong et al., 2004) and electric furnace (Menéndez et al., 2002)) in sewage sludge, the MW is superior in terms of the temperature evolution rate. Based on this observation and the fact that temperature has been reported to play an important role in the MW treatment of sludge (Banik et al., 2003, Hong et al., 2004, Shamis et al., 2008), a proper MW based full scale designated reactor will require shorter contact time than a conventional thermal reactor to achieve the same level of treatment. This in turn implies savings on time and ultimately the reactor space requirements when MW irradiation is applied.

### 4.2 Volume reduction and energy requirements

As shown in Fig. 2a and b, the MW treatment was successful in achieving over 70% weight/volume reduction in the blackwater FS within given exposure boundaries. This conforms to the results obtained by Menéndez et al. (2002) who reported 80% volume reduction in anaerobic sewage sludge. The variations in weight/volume reduction in both the 20 g and 100 g sample closely followed the temperature profiles and the drying phases discussed in Section 4.1.

Generally, the results show low weight/volume reduction in both sample sizes during the preliminary drying phase. The low weight/volume reduction can be attributed to the minimal moisture loss since the MW energy initially supplied is largely utilized for sludge temperature elevation to the boiling point and not for the water evaporation.

However, a high but relatively constant weight/volume reduction rate was observed in both samples during the essential (major) drying phase that occurred immediately after the preliminary drying phase. The duration of the entire essential drying phase within the same sample decreased with the increase in the MW power input levels. This can be attributed to the rise in the MW energy resulting from the increased power input. Moreover, the length of the essential drying phase varied with the sample size and was shorter in the smaller sample. For instance, when the 20 g sample was irradiated at 1550 W, the essential drying phase lasted until 3 min while it lasted until 7 min at the same irradiation energy for the 100 g sample. This can be explained by the total amount of water in the blackwater FS to be removed during the essential drying phase which is smaller in the small sample. Generally, the high weight/volume reduction in the essential drying phase is achieved mainly due to the removal of the free (unbound) water which requires less energy.

The third (final) drying phase was depicted by the lowest observed moisture loss in both the 20 g and 100 g sample. The low weight/volume reduction manifested in this stage is attributable to the fact that much of the water is previously evaporated in the essential drying phase and weight/volume reduction is only possible by evaporating the bound water that requires much more energy. As reported in Flaga (2005), the speed of drying at this stage decreases until it reaches a balanced hydration which is dependent on the heating temperature and the air humidity.

Similar trends in the drying phases discussed above were observed when dewatered sediment sludge was subjected to MW drying (Gan, 2000).

The energy consumption rate profiles shown in Fig. 3a and b were closely associated with the weight/volume reduction and drying phases discussed above. Differences in the energy consumption between the two sample sizes were observed, especially during the preliminary and final drying phase. The discrepancy in the energy consumption during the preliminary phase (e.g. approximately; 8.3 kWh per kg and 4 kWh per kg for 100 g and 20 g, respectively) is attributable to the fact that much of the energy at this phase is not utilized in the actual drying but in raising the sludge temperature towards boiling point. Consequently, higher energy demand is possible in the 100 g sample which has higher initial water and TS content than the 20 g sample. Furthermore, the final drying phase (only achieved here with the 1550 W) was also marked by relatively high energy demand attributable to the removal of the bound water which is more difficult to evaporate.

The lowest energy demand was observed during the essential (major) drying phase with no significant difference in the energy consumption rates between the two sample sizes. For instance, the respective energy consumption for the 100 g and 20 g sample was approximately 2.3 kWh per kg and approximately 2.5 kWh per kg. This shows that the energy supplied at this phase is mainly used in the evaporation of water, hence the similarity in the energy consumption rates between the samples. The low energy demand at this phase can be attributed to the presence of the free (unbound) water which is relatively easy to remove once the sludge is heated. Since water forms a bigger proportion of FS (approximately 88% in this case), it is evident that the essential drying phase is the most crucial phase for weight/volume reduction during the MW heating of the sludge. This implies that irradiation should be stopped at some point within the essential drying phase if the sludge is MW heated for the purpose of drying only. This is reasonable because there is no significant weight/volume reduction expected beyond this stage as manifested in the high specific energy demand in the ensuing final drying phase. The optimal point where to stop the heating within the essential drying phase will depend on the ultimate weight/volume reduction or moisture level desired and the associated energy costs. As discussed above, during the essential (major) drying phase, the specific energy requirements were relatively similar both between the two sample sizes and among the MW power levels evaluated in this study. The only difference was the contact time that was required to build up energy to the level corresponding to a certain amount of weight/volume reduction. This duration can thus be taken as the retention time of the MW reactor, a key factor in the full scale design that will affect its volume. This implies that when designing a MW reactor for applications in areas with high sludge generation rates but limited land space (e.g. the emergency camps), high MW power input with short retention time will be desirable to achieve a small reactor volume (i.e. small footprint). However, the retention time should be carefully chosen to ensure that other treatment objectives (e.g. pathogen reduction) are achieved.

Despite the fairly low energy demand, it is notable that the specific energy consumptions attained in the essential drying phase here are higher than in other drying methods, especially the convective and conductive industrial driers which according to Bennamoun et al. (2013) vary between 0.7–1.4 and 0.8–1.0 kWh, respectively, per kg of evaporated water. The cause of this disparity may be twofold. Firstly, the specific energy consumptions as reported by Bennamoun et al. (2013) are based on sewage sludge whose properties may differ from that of the blackwater FS, especially the viscosity which may hinder the microwaves penetration capacity (Hong et al., 2004). Secondly, the lack (in the unit used in the this study) of some important design aspects that are found in the industrial driers such as customized ventilation for moisture extraction may also be a major contribution to the disparities. Customizing the unit's design for maximum vapor extraction during the treatment, especially during the essential drying phase, may possibly reduce the energy demand. Furthermore, the results obtained here are only preliminary, and thus more research is needed to optimize the system, increase efficiency and reduce energy input. More tests with different kinds of sludge are also needed.

### 4.3 Bacterial reduction

The results shown in Fig. 4, Fig. 5 demonstrate that MW irradiation was very rapid and effective in the reduction of *E. coli* in blackwater FS. This observation is consistent with those from previous studies (Border and Rice-Spearman, 1999, Lamb and Siores, 2010), although these studies used different sample material. The destruction of *E. coli* during the MW irradiation treatment can be attributed to both the non-thermal (electromagnetic radiation) and the thermal (temperature) effects. The electromagnetic radiation effect has been identified a factor in pathogen destruction in MW treatment (Banik et al., 2003, Hong et al., 2004, Shamis et al., 2008), but the temperature effect is considered the main mechanism for which 70 °C is identified as the minimum temperature essential for complete bacterial reduction (Hong et al., 2004, Tyagi and Lo, 2012, Valero et al., 2014). As observed here, for any given power level, the contact time necessary for complete bacterial reduction will vary with the amount of material treated. This is clearly illustrated in the two sample sizes evaluated. For instance, *E. coli* was detected when the 100 g sample was irradiated at 465 W for 1 min (MW energy = 7.75 Wh, temp. = 51 °C) while none was detected in the 20 g sample treated under similar operational conditions. The disparity in reduction between these two sets of samples can be attributed to the observed differences in their final temperature (i.e. 71 °C in 20 g sample and 51 °C in 100 g sample (Fig. 5a and b)). As discussed in Section 4.1, temperature increases in the sludge are largely dependent on the water content; larger samples will require longer contact time to achieve the lethal temperature necessary for complete reduction. Despite 70 °C being considered the minimum temperature lethal to pathogenic bacteria, live cells were still detected when this temperature was attained in the 20 g sample irradiated at 1550 W for 10 s (see Fig. 5a). This implies that upon attaining the minimum lethal temperature, the microorganisms need to be exposed for an additional minimum duration to ensure complete die off. Therefore, when the aim of treatment is only to destruct bacteria in the sludge, the total contact time for the entire process will be the sum total of the time taken to attain the lethal temperature level and the additional minimum exposure time required for complete die off. How fast this is achieved will depend on the MW power level applied and the amount of material treated. Furthermore, it is evident from the results here that complete bacteria (*E. coli*) reduction can be achieved over short contact times when the material is irradiated at higher MW power levels. High power input results in rapid escalation of the temperature which is a key contributor to bacterial die off. Thus when targeting complete reduction in a MW based reactor, it is desirable to use high MW power to realize shorter retention time and smaller reactor footprints. This is desirable especially in the emergency situations where land space is often a major constraint. Moreover, comparing the MW energy requirements for weight/volume reduction and the *E. coli* reduction, it can be inferred that more power is required to realize substantial drying than the bacterial reduction. In the 100 g sample, for instance, while over 180 Wh was required to attain a weight/volume reduction of approximately 70% (Fig. 3b), complete *E. coli* reduction was achieved at 23 Wh (Fig. 4b).

### 4.4 Organic stabilization

From the results shown in Fig. 6a and b, it is evident that MW treatment was not successful in VS/TS reduction in both the 20 g and 100 g sample. After the MW treatment, the final VS/TS ratios attained in each sample size were higher than the 60% recommended by the European Environment Agency (Bresters et al., 1997) as the reference for the organically stable sludge. Thus, under the test conditions carried out in this study, organic stabilization of the blackwater FS was not achieved. This is expected since the highest temperature attained during the irradiation was approximately 127 °C (i.e. 20 g sample, 1550 W, 4 min contact time) which is much lower than the 550 °C normally applied for VS ignition in the gravimetric method (SM-2540E) (APHA, 1995) during the VS measurements. Nevertheless, if the MW technology is aimed at specific situations such as the FS treatment in areas of intensive sanitation facility usage, e.g. emergency situations, the organic sludge instability may not be a major concern provided that pathogens are fully inactivated and the public health risk is reduced. Furthermore, as demonstrated in this study, the sludge weight/volume can be largely reduced by the MW irradiation making it possible to cost-efficiently treat it further (for organic stabilization) by less costly options such as composting, sludge drying beds, anaerobic digestion, etc., that also promote resource recovery.

## 5 Conclusions and recommendations

The MW treatment was not only able to achieve over 70% weight/volume reduction but also a complete reduction of the pathogenic bacteria indicator (*E. coli*) in the sludge. However, under the experimental conditions evaluated in this study, the MW treatment expectedly, did not yield substantial organic stabilization of the sludge. Nevertheless, with further developments, the MW technology can be considered a promising option for the rapid treatment of fresh FS. Particularly, testing for other microorganisms such as helminth (e.g. *Ascaris lumbricoides*) eggs, enteroviruses, etc., is desired to assess the MW capability to achieve complete FS sanitization. Further research is also required to develop a pilot-scale MW based reactor unit and test with FS from intensively used sanitation facilities such as toilets in emergencies or similar situations.

## Figures and Tables

**Table 1:** Physico-chemical characteristics of the fresh blackwater and the blackwater FS.

Parameter	Blackwater	Blackwater FS
Value	Value
Water content (%)	98.6	88
Total solids, %	1.4	12
Volatile solids, %	1.3	10.7
pH	6.9–7	6.7
Total COD, TCOD (mg/g TS)	1643	1344
*E. coli* (CFU/g TS)	2.3 × 10^6^	4.0 × 10^8^

## References

[bb0005] APHA (1995). Standard methods.

[bb0010] Banik S., Bandyopadhyay S., Ganguly S. (2003). Bioeffects of microwave—a brief review. Bioresour. Technol..

[bb0015] Bennamoun L., Arlabosse P., Léonard A. (2013). Review on fundamental aspect of application of drying process to wastewater sludge. Renew. Sust. Energ. Rev..

[bb0020] Border B.G., Rice-Spearman L. (1999). Microwaves in the laboratory: effective decontamination. Clin. Lab. Sci..

[bb0025] Bresters A.R., Coulomb I., Matter B., Saabye A., Spinosa L., Utvik Å.Ø. (1997). Management approaches and experiences: sludge treatment and disposal.

[bb0030] Byamukama D., Kansiime F., Mach R.L., Farnleitner A.H. (2000). Determination of *Escherichia coli* contamination with chromocult coliform agar showed a high level of discrimination efficiency for differing fecal pollution levels in tropical waters of Kampala, Uganda. Appl. Environ. Microbiol..

[bb0035] Eskicioglu C., Terzian N., Kennedy K.J., Droste R.L., Hamoda M. (2007). Athermal microwave effects for enhancing digestibility of waste activated sludge. Water Res..

[bb0040] Fidjeland J., Magri M.E., Jönsson H., Albihn A., Vinnerås B. (2013). The potential for self-sanitisation of faecal sludge by intrinsic ammonia. Water Res..

[bb0045] Flaga A., Plaza E., Levlin E. (2005). Sludge drying. Proceedings of Polish-Swedish Seminars Integration and Optimisation of Urban Sanitation Systems, Cracow, Poland.

[bb0050] Gan Q. (2000). A case study of microwave processing of metal hydroxide sediment sludge from printed circuit board manufacturing wash water. Waste Manag..

[bb0055] Haque K.E. (1999). Microwave energy for mineral treatment processes—a brief review. Int. J. Miner. Process..

[bb0060] Hong S.M., Park J.K., Lee Y.O. (2004). Mechanisms of microwave irradiation involved in the destruction of fecal coliforms from biosolids. Water Res..

[bb0065] Hong S.M., Park J.K., Teeradej N., Lee Y.O., Cho Y.K., Park C.H. (2006). Pretreatment of sludge with microwaves for pathogen destruction and improved anaerobic digestion performance. Water Environ. Res..

[bb0070] Ingallinella A.M., Sanguinetti G., Koottatep T., Montanger A., Strauss M. (2002). The challenge of faecal sludge management in urban areas — strategies, regulations and treatment options. Water Sci. Technol..

[bb0075] Katukiza A.Y., Ronteltap M., Niwagaba C.B., Foppen J.W.A., Kansiime F., Lens P.N.L. (2012). Sustainable sanitation technology options for urban slums. Biotechnol. Adv..

[bb0080] Lamb A.S., Siores E., Anand S.C., Kennedy J.F., Miraftab M., Rajendran S. (2010). A review of the role of microwaves in the destruction of pathogenic bacteria. Medical and Healthcare Textiles.

[bb0085] Lin Q.H., Cheng H., Chen G.Y. (2012). Preparation and characterization of carbonaceous adsorbents from sewage sludge using a pilot-scale microwave heating equipment. J. Anal. Appl. Pyrolysis.

[bb0090] Lopez-Vazquez C.M., Dangol B., Hooijmans C.M., Brdjanovic D., Strande L., Ronteltap M., Brdjanovic D. (2014). Co-treatment of faecal sludge in municipal wastewater treatment plants. Faecal Sludge Management —Systems Approach Implementation and Operation.

[bb0095] Menéndez J.A., Domínguez A., Inguanzo M., Pis J.J. (2005). Microwave-induced drying, pyrolysis and gasification (MWDPG) of sewage sludge: vitrification of the solid residue. J. Anal. Appl. Pyrolysis.

[bb0100] Menéndez J.A., Inguanzo M., Pis J.J. (2002). Microwave-induced pyrolysis of sewage sludge. Water Res..

[bb0105] Oxfam (2011). Urban WASH Lessons Learned From Post-earthquake Response in Haiti, Oxford, UK. http://policy-practice.oxfam.org.uk/publications/urban-wash-lessons-learned-from-post-earthquake-response-in-haiti-136538.

[bb0110] Remya N., Lin J.-G. (2011). Current status of microwave application in wastewater treatment—a review. Chem. Eng. J..

[bb0115] Ronteltap M., Dodane P.-H., Bassan M., Strande L., Ronteltap M., Brdjanovic D. (2014). Overview of treatment technologies. Faecal Sludge Management — Systems Approach Implementation and Operation.

[bb0120] Sangadkit W., Rattanabumrung O., Supanivatin P., Thipayarat A. (2012). Practical coliforms and *Escherichia coli* detection and enumeration for industrial food samples using low-cost digital microscopy. Procedia Eng..

[bb0125] Shamis Y., Taube A., Shramkov Y., Mitik-Dineva N., Vu B., Ivanova E.P. (2008). Development of a microwave treatment technique for bacterial decontamination of raw meat. Int. J. Food Eng..

[bb0130] Strande L., Strande L., Ronteltap M., Brdjanovic D. (2014). The global situation. Faecal Sludge Management — Systems Approach Implementation and Operation.

[bb0135] Tang B., Yu L., Huang S., Luo J., Zhuo Y. (2010). Energy efficiency of pre-treating excess sewage sludge with microwave irradiation. Bioresour. Technol..

[bb0140] Tappero J.W., Tauxe R.V. (2011). Lessons learned during public health response to cholera epidemic in Haiti and the Dominican Republic. Emerg. Infect. Dis..

[bb0145] Thostenson E.T., Chou T.W. (1999). Microwave processing: fundamentals and applications. Compos. A: Appl. Sci. Manuf..

[bb0150] Tyagi V.K., Lo S.-L. (2012). Enhancement in mesophilic aerobic digestion of waste activated sludge by chemically assisted thermal pretreatment method. Bioresour. Technol..

[bb0155] Tyagi V.K., Lo S.-L. (2013). Microwave irradiation: a sustainable way for sludge treatment and resource recovery. Renew. Sust. Energ. Rev..

[bb0160] Valero A., Cejudo M., García-Gimeno R.M. (2014). Inactivation kinetics for *Salmonella enteritidis* in potato omelet using microwave heating treatments. Food Control.

[bb0165] Watson J.T., Gayer M., Connolly M.A. (2007). Epidemics after natural disasters. Emerg. Infect. Dis..

[bb0170] WHO, Lorna F., Jamie B. (2001). Excreta-related infections and the role of sanitation in the control of transmission. Water Quality: Guidelines, Standards & Health: Assessment of Risk and Risk Management for Water-related Infectious Disease.

[bb0175] WHO (2006). Guidelines for the safe use of wastewater, excreta and greywater.

[bb0180] Wojciechowska E. (2005). Application of microwaves for sewage sludge conditioning. Water Res..

[bb0185] Yu Q., Lei H., Li Z., Li H., Chen K., Zhang X., Liang R. (2010). Physical and chemical properties of waste-activated sludge after microwave treatment. Water Res..

